# A Large Expansion of the *HSFY* Gene Family in Cattle Shows Dispersion across Yq and Testis-Specific Expression

**DOI:** 10.1371/journal.pone.0017790

**Published:** 2011-03-07

**Authors:** Christine K. Hamilton, Tamas Revay, Robin Domander, Laura A. Favetta, W. Allan King

**Affiliations:** Department of Biomedical Sciences, University of Guelph, Guelph, Ontario, Canada; Temasek Life Sciences Laboratory, Singapore

## Abstract

Heat shock transcription factor, Y-linked (*HSFY*) is a member of the heat shock transcriptional factor (HSF) family that is found in multiple copies on the Y chromosome and conserved in a number of species. Its function still remains unknown but in humans it is thought to play a role in spermatogenesis. Through real time polymerase chain reaction (PCR) analyses we determined that the *HSFY* family is largely expanded in cattle (∼70 copies) compared with human (2 functional copies, 4 *HSFY*-similar copies). Unexpectedly, we found that it does not vary among individual bulls as a copy number variant (CNV). Using fluorescence *in situ* hybridization (FISH) we found that the copies are dispersed along the long arm of the Y chromosome (Yq). *HSFY* expression in cattle appears restricted to the testis and its mRNA correlates positively with mRNA markers of spermatogonial and spermatocyte cells (*UCHL1* and *TRPC2*, respectively) which suggests that *HSFY* is expressed (at least in part) in early germ cells.

## Introduction

The Y chromosome was originally thought to be devoid of functional genes because of its small size and heterochromatic nature [1]. This theory has since been disproven and now about 78 protein-encoding genes have been assigned to this chromosome in humans, most of which are involved in male growth, development and spermatogenesis [2], [3]. The Y chromosome is unique in that the majority of its length does not pair with the X chromosome during meiosis to undergo homologous recombination [2]. This region is known as the male specific region or MSY [2]. The MSY is enriched with multi-copied genes and copy number variants (CNVs) [2], [4]. CNVs are DNA segments of at least 1 kb in size that can vary in copy number among individuals through deletions and duplications and in many cases this variation has been linked to gene expression and phenotype [5]–[8]. Although there is still a lack of sequence data for the MSY, it has been fully sequenced in both human and chimpanzees and even between these closely related species, it shows enormous (and somewhat unexpected) diversity [9]. This diversity manifests itself in gene structure, content and number of gene copies.

An example of a multi-copied MSY gene is *HSFY*. It is a member of the heat shock transcriptional factor (HSF) family which are able to bind heat shock elements on heat shock protein (HSP) genes to regulate gene expression [10]. HSPs are important mediators of stress and act as molecular chaperones to regulate cellular homeostasis and promote cell survival in conditions that would otherwise be fatal [11], [12].

*HSFY* contains a heat shock factor type A DNA-binding domain that is similar to that found in other HSF genes, including the X-homologue (LW-1) [13]–[15]. Its three-dimensional conformation, however, is altered so it is unknown if *HSFY* can act as a transcriptional regulator [13], [14]. Its expression is reported to be mainly testis-specific in humans [15]. More specifically, *HSFY* expression seems restricted to Sertoli and spermatogenic cells [14]. It is likely that *HSFY* is involved in spermatogenesis but its exact function remains unknown [14]–[17].

The copy number of *HSFY* appears to vary between species. It has been measured in humans and felines and is present in 2 and about 8 copies, respectively [2], [18]. *HSFY* orthologs have been found in a variety of other species including mouse, rat, rhesus macaque, and dogs and appears to be conserved, however, the gene copy number in these species has not yet been characterized [16], [19]. Cattle have an *HSFY* ortholog (*HSFY2*) and it has been hypothesized that it is present in multiple copies but this theory remains speculative and no attempt has been made to characterize the number of copies [20].

The purpose of this study was to determine the exact genomic copy number of the *HSFY* family in different individuals, determine its chromosomal location and to determine its expression pattern in Canadian Holstein cattle. We found that bulls contain around 70 copies of the *HSFY* gene which are dispersed along the long arm of the Y chromosome and we provide evidence that *HSFY* expression is testis-specific.

## Methods

### HSFY gene analysis and sequencing

A comprehensive search of the sequence database on the NCBI website was carried out in order to find and compare *HSFY* orthologs among different species. Structural similarities between deduced amino acid sequences among human *HSFY* sequences (hHSFY1: NP_149099.2; hHSFY2: NP_714927.1), as well as mouse (mHSFYL: NP_081937.1), rat (NP_001012132.1), rhesus macaque (ACL51668.1), cat (NP_001035212.1), and bovine (NP_001070474.1) were determined by multiple sequence alignments carried out using CLUSTAL W software [21]. The current bovine *HSFY* sequence (*HSFY2*: NW_001498001.1) is based on the Hereford breed of cattle and was assembled as part of the bovine genome project (Assembly Btau_4.2) [22]. We deduced the *HSFY* gene sequence in the Holstein breed by sequencing the PCR products that were generated throughout the study (as described below) and the resultant sequence was deposited into GenBank with accession number JF281100. Breed specific differences were analyzed by comparing the predicted Holstein amino acid sequence with the current predicted *HSFY* amino acid sequence derived from the Hereford breed using CLUSTAL W software.

### Sample collection and DNA/cDNA preparation

A variety of tissues (blood, heart, kidney, liver, lung, ovary, testis) were obtained from a bank of tissues (L'Alliance Boviteq Inc., St Hyacinthe, Quebec, Canada) collected from a slaughtered Holstein heifer and from 24 slaughtered Holstein bulls. DNA was extracted using methods previously described [23]. Briefly, DNA was extracted from blood samples using standard phenol-chloroform methods. Total mRNA was extracted from the remaining tissues using a RNeasy Mini kit (QIAGEN Inc.) and treated with DNAse I (TURBO DNA-free, Ambion Inc.) following manufacturers' instructions. 1 µg of total mRNA was reverse transcribed with Superscript II reverse transcriptase (Invitrogen Canada Inc.) using oligo(dT) primers (Invitrogen Canada Inc.) according to manufacturers' instructions for all tissue samples. For each bull, the fertility status was obtained, as measured by 56-day non-return rates (NRRs). A 56-day NRR refers to the proportion of cows inseminated that were not reported to return to oestrus after 56 days and is used as an estimate of conception [24].

### Quantitative PCR

**Figure 1:**
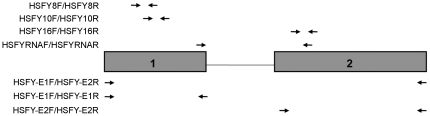
Schematic bovine *HSFY* gene and targeted regions of the primers used for the PCR experiments. The numbered boxes represent the gene exons and the adjoining line represents the intron. Primer sets HSFY8F/HSFY8R (111 bp), HSFY10F/HSFY10R (113 bp), and HSFY16F/HSFY16R (102 bp) were used to measure *HSFY* copy number with real time PCR and target individual exons. Primer set HSFYRNAF/HSFYRNAR (215 bp) was designed specifically for mRNA amplification: HSFYRNAF targets exon 1 and HSFYRNAR targets exon 2 and together they span an intron to avoid co-amplification of residual DNA. The three primer sets shown below the schematic *HSFY* gene were used to create PCR probes for the fluorescence *in situ* hybridization (FISH) experiments and they target the full gene (including both exons and the intron: HSFY-E1F/HSFY-E2R, 1686 bp), exon 1 (HSFY-E1F/HSFY-E1R, 515 bp) and exon 2 (HSFY-E2F/HSFY-E2R, 752 bp). Primer sequences are listed in Table 1.

The gene sequences used to design all primer sets were accessed from the sequence database on the NCBI website and their accession numbers are listed in Table 1. Primers were designed for these sequences using Primer3 software [25]. Three sets of primers (designated HSFY8, HSFY10, HSFY16) targeting different regions of *HSFY* were used relative to the single copy reference gene, *SRY*, to measure *HSFY* copy number (Figure 1, Table 1). A separate set of primers targeting *HSFY* mRNA (designated HSFYRNA) was designed to span an intron to ensure cDNA specificity (Figure 1, Table 1). Primers targeting the mRNA specific markers for spermatogonia and spermatocytes, *UCHL1* and *TRPC2*, respectively and the reference gene, *GAPDH*, were also used in the mRNA experiments [26], [27]. To ensure male-specificity, both male and female DNA were run with the *HSFY* primers as well as an autosomal gene, *ZAR1* (Table 1). Melting curve analyses, gel electrophoresis and PCR product sequencing (University of Guelph Laboratory Services, Guelph, ON, Canada) were used to verify amplification of the correct target genes.

**Table 1:** Primer sequences used for real time PCR experiments and FISH probe preparation.

Primer name	Primer sequences (5′→3′)	Size^a^	GenBank accession number	Gene name	Gene symbol	Annealing temp (°C)
GAPDHF	TTCCTGGTACGACAATGAATTTG	153	NM_001034034.1	glyceraldehyde-3-phosphate dehydrogenase	*GAPDH*	66
GAPDHR	GGAGATGGGGCAGGACTC					
HSFY8F	CAGAAACCTCCGTTAGATCTTC	111	NW_001498001.1	heat shock transcription factor, Y linked 2	*HSFY2*	67
HSFY8R	TCACAAGATCCTCAGACAAAGC					
HSFY10F	TGCTTTTCAGGCTTTGTCTG	113	NW_001498001.1	heat shock transcription factor, Y linked 2	*HSFY2*	67
HSFY10R	CCAAAGTTTTGTGGAAATGTGA					
HSFY16F	ATTCTGGACTGATGGTGGAG	102	NW_001498001.1	heat shock transcription factor, Y linked 2	*HSFY2*	67
HSFY16R	ACGAGTTGCCTGTCTCTTGC					
HSFYRNAF	GCTTCACTACCTGTCTTTCTGG	215	NM_001077006.1	heat shock transcription factor, Y linked 2	*HSFY2*	66
HSFYRNAR	TCATCTTCGTTGACACTTGC					
HSFY-E1F	ATTCAAGATGGGCCTCCTAAG	1686	NW_001498001.1	heat shock transcription factor, Y linked 2	*HSFY2*	65
HSFY-E2R	GTTGACATCTCCCAAAGTAACG					
HSFY-E1R^b^	GCCAATAAGTGAAAATGTTGGA	515	NW_001498001.1	heat shock transcription factor, Y linked 2	*HSFY2*	58
HSFY-E2F^c^	AATCCAAATTTCAAAAGAGGTCA	752	NW_001498001.1	heat shock transcription factor, Y linked 2	*HSFY2*	58
SRYF	CCAATTAAGCCGGTCACAGT	162	NW_001505546.1	sex determining region Y	*SRY*	67
SRYR	GCACAAGAAAGTCCAGGCTC					
TRPC2F	AAGATTGAGGACGCTGCTGACG	180	NM_174477.3	transient receptor potential channel 2	*TRPC2*	66
TRPC2R	TTTGCAGCAGAGGTGGCAACAG					
UCHL1F	ACCCCGAGATGCTGAACAAAG	323	NM_001046172.1	ubiquitin carboxyl-terminal esterase L1	*UCHL1*	66
UCHL1R	CCCAATGGTCTGCTTCATGAA					
ZAR1F	AAGTGCCTATGTGTGGTGTG	191	NC_007304.4	zygote arrest 1	*ZAR1*	67
ZAR1R	CAGGTGATATCCTCCACTCG					

a PCR product size in base pairs,

b primer used in combination with HSFY-E1F,

c with HSFY-E2R.

The table shows the primer name, primer sequences, the predicted PCR product size (base pairs, bp), GenBank accession number of the sequence that was used as the target for primer design, gene name and symbol, and the unique annealing temperature (°C) that was used for the PCR amplification.

1 ng of DNA and 50 ng of the reverse transcription product (cDNA) were analyzed by real time PCR as described previously [23]. Briefly, the real time PCR was performed using a LightCycler 1.5 apparatus (Roche Diagnostics) and a LightCycler Fast Start DNA Master SYBR Green I kit (Roche Diagnostics). The primer and MgCl_2_ concentrations were 0.5 µM and 3 mM, respectively. Samples were run in triplicate. The DNA samples were analyzed with two separate PCR runs that were averaged to minimize variability.

A calibrator sample was included in each run to minimize inter-run variability [28]. Primer efficiencies were measured according to the equation E = 10^[−1/slope]^ [29]. Normalized ratios were determined for all runs using the 2^−ΔΔCT^ method (PE Applied Biosystems, Perkin Elmer, Forster City, CA) for DNA runs and the Pfaffl method of quantification for the cDNA runs [30]. *HSFY* copy number of the calibrator sample was determined with the 2^−ΔCT^ method using three sets of primers and averaged.

For statistical analyses all data first underwent the Kolmogorov-Smirnov test of normality. To determine differences between groups, we performed one way analysis of variance (ANOVA). All correlations were analyzed with a Pearson's correlation coefficient. Data is reported as the mean ± SEM unless otherwise stated. A p-value of less than 0.05 is considered significant. The data was analyzed using GraphPad Prism (version 5.02) software (GraphPad Software, Inc., San Diego, CA).

### Fluorescence in situ hybridization (FISH)

Metaphase chromosome spreads were prepared from a bull's peripheral blood sample cultivated in RPMI1640 (Invitrogen Canada Inc.) containing 10% FBS (Invitrogen Canada Inc.), 2% L-Glutamine (Sigma-Aldrich), 1% PenStrep (Invitrogen Canada Inc.), 1.5% Phytohemagglutinin (Invitrogen Canada Inc.) for 3 days. KaryoMax Colcemid (0.025 µg/ml, Invitrogen Canada Inc.) was added to the culture for the last 45 minutes. Slides were prepared from the hypotonized and MeOH/AcOH (3/1) fixed suspensions according to standard cytogenetic techniques and aged a minimum of 3 days before using for FISH. Three different biotinilated hybridization probes were prepared by PCR. The resulting PCR product were sequenced to confirm specificity of the primers. Primers were designed to anneal at the borders of both exons and were used in different combinations to amplify the whole *HSFY* gene (HSFY-E1F/HSFY-E2R) or exon1 (HSFY-E1F/HSFY-E1R) or exon 2 (HSFY-E2F/HSFY-E2R) selectively (Figure 1, Table 1). PCR mixes contained 80 ng bull genomic DNA, 2.5 Units AmpliTaq Gold DNA Polymerase (Applied Biosystems), 1×PCR Buffer II, 1.5 mM MgCl_2_, 200 µM each dATP, dCTP, dGTP, 133 µM dTTP, 67 µM biotin-11-dUTP (Metkinen) and 0.5 µM of each primer. For the primer sets that amplified the whole *HSFY* gene (HSFY-E1F/HSFY-E2R), the PCR mixture underwent the following thermal profile: 95°C, 10 min; 30×(94°C, 45 sec; 65°C, 45 sec; 72°C, 105 sec), 72°C, 10 min. For the other two primer sets which amplified exon 1(HSFY-E1F/HSFY-E1R) and exon 2 (HSFY-E2F/HSFY-E2R) the PCR mixture underwent the following thermal profile: 95°C, 10 min; 30×(94°C, 45 sec; 58°C, 45 sec; 72°C, 45 sec), 72°C, 10 min. All PCR reactions were performed with a MJ Research PTC-200 Thermo Cycler. 6 µl PCR product was added to the hybridization mixture, composed of 50% (v/v) formamide (Fisher), 10% (w/v) dextran-sulphate (Sigma-Aldrich), 2×SSC and denatured at 72°C for 10 min before applying to the slide.

The FISH experiments were performed with standard protocols, briefly: slides were treated with pepsin, dehydrated in ethanol series, denatured at 72°C for 1 minute in 70% formamide (Fisher)/2×SSC, then quenched in ice-cold ethanol. Hybridization occurred at 37°C overnight, followed by 5 minute washes in 2×SSC, 0.2×SSC twice at 37°C and PBS/0.5% Tween 20 at room temperature. Biotinylated signals were visualized by a single layer of FITC-avidin (Vector, 1∶400 in PBS/0.5% Blocking Reagent, (Roche)) incubated for 30 minutes at 37°C and washed by 3×(PBS/0.5% Tween 20) at room temperature. Chromosomes were counterstained with DAPI (Sigma-Aldrich) and slides were mounted with Vectashield (Vector). Images were captured using a Leica DM5500B fluorescence microscope (Leica), equipped with a Retiga Exi Fast (QImaging) cooled digital camera.

## Results

### HSFY sequencing

A comprehensive search of the NCBI website revealed a bovine *HSFY* ortholog, *HSFY2* (Entrez Gene ID: 767933). The genomic sequence of this gene was published as part of the bovine genome project (based on Btau_4.2; NW_001498001.1) and derived from the Hereford breed of cattle [22]. The complete mRNA coding sequence (NM_001077006.1) and predicted protein sequence (NP_001070474.1) were also available for Hereford cattle in the database.

**Figure 2:**
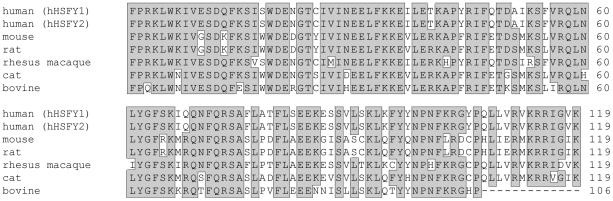
Sequence alignment of the conserved DNA-binding domain in various species. The conserved heat shock factor (HSF) type DNA binding-domain shows a high degree of sequence homology among different species. Amino acids conserved between at least 5 proteins are marked with shaded boxes. The accession numbers for the proteins deposited in the sequence database on the NCBI website are as follows: hHSFY1 NP_149099.2; hHSFY2 NP_714927.1; mHSFYL NP_081937.1; rat NP_001012132.1; rhesus macaque ACL51668.1; cat NP_001035212.1; bovine (Hereford) NP_001070474.1.

*HSFY* protein sequence alignments had been previously performed in human, mouse and cow, however, detailed percent homologies had not yet been described [16]. For this study, the alignments were expanded to include both copies of *HSFY* in human (hHSFY1 and hHSFY2) as well as the *HSFY* sequences of mouse, rat, rhesus macaque, cat and cow. Comparisons of sequence homologies for the complete *HSFY* protein as well as the conserved HSF-type DNA-binding domain for all species are presented in Table 2. Figure 2 shows the alignment results of the conserved HSF-type DNA-binding domain among different species. Both copies of human *HSFY* (*HSFY1* and *HSFY2*) were found to encode identical proteins (100% homology). Overall, cattle show 51% sequence homology with human *HSFY* and within the conserved DNA-binding domain the homology is increased to 72%. It showed its highest sequence identity with cat, with percent homologies of 58% and 81%, for the complete protein and HSF-type DNA-binding domain, respectively.

**Table 2:** Comparison of the percent homologies (%) of the complete *HSFY* protein (A) and HSF-type DNA-binding domain (B), for all species analyzed.

	human (hHSFY2)		mouse (mHSFYL)		rat		rhesus macaque		cat		bovine	
	A	B	A	B	A	B	A	B	A	B	A	B
human (hHSFY1)	100	100	43	71	44	71	81	87	64	78	51	72
human (hHSFY2)	-	-	43	71	44	71	81	87	64	78	51	72
mouse (mHSFYL)			-	-	95	100	43	69	47	80	40	76
rat					-	-	43	69	46	80	39	76
rhesus macaque							-	-	58	73	43	69
cat									-	-	58	81

In all cases, the percent homology was highest at the conserved HSF-type DNA-binding domain with the exception of an analysis of the two human HSFY copies which showed that both the complete protein sequence and conserved region were identical (100% homology). Bovine showed 51% and 72% homology with human HSFY for the complete protein and conserved domain, respectively. Bovine HSFY was most closely related to cat HSFY with a complete protein homology and conserved domain homology of 58% and 81%, respectively. The accession numbers for the proteins found in the NCBI database are as follows: human hHSFY1: NP_149099.2; hHSFY2: NP_714927.1, mouse NP_081937.1, rat NP_001012132.1, rhesus macaque ACL51668.1, cat NP_001035212.1, bovine NP_001070474.1.

**Figure 3:**
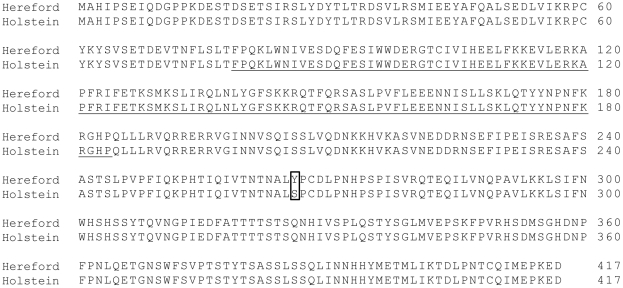
Comparison of the predicted amino acid sequences of *HSFY* in Hereford and Holstein cattle. The mRNA sequences are 99% homologous and differ by only one base pair (c.1192A>C). This missense substitution mutation (p.*Tyr266Ser*) results in an amino acid change from tyrosine (Y) in Hereford to serine (S) in Holstein in the predicted protein sequence (shown by the box). This mutation is not found in the conserved HSF-type DNA-binding domain which is shown by the underlined sequence.

The complete *HSFY* gene sequence was deduced in Holstein and submitted to GenBank (accession number: JF281100). The Holstein *HSFY* genomic sequence was found to differ with the Hereford *HSFY* sequence by two nucleotides (c.56G>A, c.1192A>C). The published Hereford mRNA sequence, however, also shows the first discrepancy with the Hereford genomic sequence (c.56G>A) and therefore this one mismatch likely represents a genomic sequencing error. Our Holstein genomic sequence matches the Hereford mRNA sequence at this position. The second point mutation we found (c.1192A>C) is consistently different from both the Hereford genomic and mRNA sequences currently deposited on the NCBI database. This mutation results in a substitution from tyrosine (Hereford) to serine (Holstein) at position 266 of the predicted *HSFY* protein (p.*Tyr266Ser*). An alignment of the predicted amino acid sequences for both Hereford and Holstein cattle is shown in Figure 3.

### HSFY gene copy number

**Figure 4:**
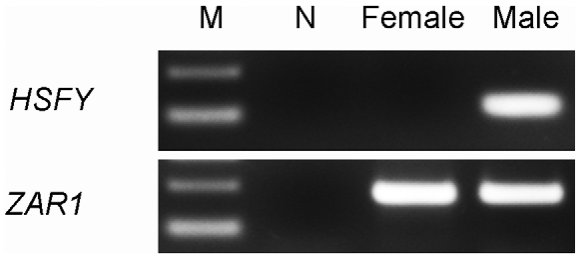
Gel electrophoresis of the real time PCR product of both male and female DNA samples for *HSFY* and *ZAR1*. *HSFY* shows male-specific amplification indicating that the target genes are on the Y chromosome. Zygote arrest 1 (*ZAR1*) is the autosomal control gene which shows amplification in both the male and female sample. N) Negative control M) 100 bp ladder – the top and bottom band represent 200 and 100 bp, respectively (Invitrogen Canada Inc.).

Primer efficiencies were tested and were not found to be significantly different among targets (HSFY8, HSFY10, HSFY16) and the reference gene (SRY). We used the *HSFY* primers on both male and female DNA samples and as expected we found amplification in the male samples only whereas *ZAR1*, which is an autosomal gene, was present in both (Figure 4).

**Figure 5:**
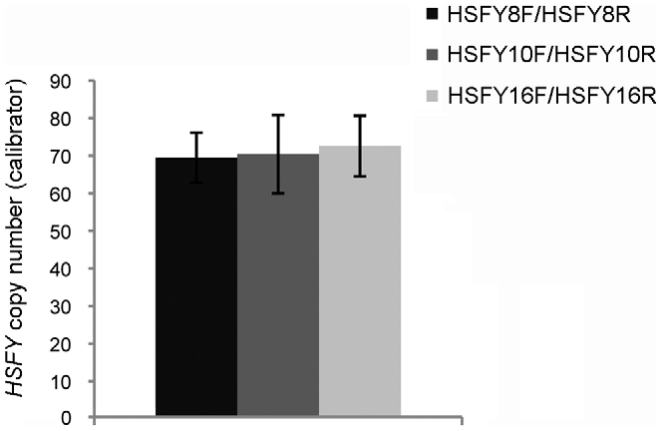
*HSFY* copy number in the calibrator sample measured by real time PCR with three different primers. There is no significant difference in mean *HSFY* copy number as measured by three different primer sets (HSFY8: HSFY8F/HSFY8R; HSFY10: HSFY10F/HSFY10R, and HSFY16: HSFY16F/HSFY16R) which target different regions of the gene. *HSFY* copy numbers for primer sets HSFY8, HSFY10 and HSFY16 were based on 20, 4 and 3 separate real time PCR runs, respectively.

Three separate *HSFY* primers targeting different regions of the gene were used to measure *HSFY* copy number in the calibrator sample. There were no significant differences in *HSFY* copy number measured with the three different primers (Figure 5). The average *HSFY* copy number of the calibrator sample was 70.8±0.9 copies. We selected the HSFY8 primers to analyze the full sample set (n = 24) and found that *HSFY* copy number did not vary significantly from bull to bull (Table 3). The average *HSFY* copy number amongst all bulls measured was 73.3±0.8 and this value did not differ significantly with that of the calibrator. All data was normally distributed. The fertility status of the bulls ranged from an NRR of 49.6%–77.3% with an average of 65.9% but showed no significant correlation to *HSFY* copy number or mRNA levels (Table 3).

**Table 3:** *HSFY* copy number (n = 24), *HSFY* mRNA levels (n = 22) and percent 56-day non-return rate (NRR) for all bulls.

Bull	*HSFY* copy number ± SEM	Relative *HSFY* mRNA ± SEM	56-day non-return rate (NRR) (%)
1	75.7±8.5	0.851±0.078	66.1
2	73.2±3.2	0.572±0.046	66.9
3	69.1±9.0	0.490±0.016	70.0
4	77.5±17.0	0.395±0.007	49.6
5	67.5±5.6	0.283±0.013	58.0
6	67.5±4.1	0.339±0.010	72.6
7	73.5±1.5	N/A	74.5
8	69.1±4.3	0.295±0.014	70.1
9	80.8±0.4	0.653±0.060	58.8
10	68.4±11.1	0.568±0.031	70.0
11	75.9±13.9	0.378±0.014	57.3
12	69.4±1.9	0.285±0.004	67.1
13	74.8±10.2	0.506±0.006	69.0
14	71.5±16.5	0.308±0.007	71.0
15	74.3±6.9	0.834±0.028	66.1
16	72.7±0.3	0.373±0.002	66.4
17	76.5±10.6	0.447±0.001	65.0
18	75.7±7.9	0.762±0.013	60.0
19	73.1±0.7	0.391±0.010	57.4
20	84.6±21.3	0.278±0.016	73.0
21	73.0±6.9	0.515±0.006	74.8
22	73.4±3.8	0.706±0.042	64.0
23	72.0±7.1	0.500±0.027	57.0
24	70.7±5.7	N/A	77.3
Average	73.3±0.8	0.489±0.038	65.9

N/A = testis sample not available.

There were no significant differences in mean HSFY copy number among bulls and the average was 73.3±0.8 copies. HSFY mRNA levels did vary significantly among bulls (p<0.0001) with an average of 0.489±0.038 relative to GAPDH. The 56-day non-return rates, a measure of field fertility, ranged from 49.6%–77.3%, with an overall average of 65.9%.

### Fluorescence in situ hybridization

**Figure 6:**
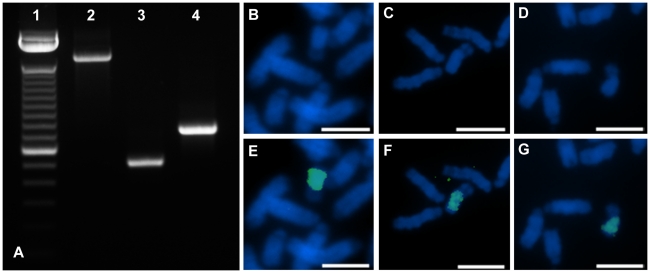
Gel electrophoresis of hybridization probes (A) and flourescence *in situ* hybridization (FISH) analysis (B–G). A) The largest intense band in lane 1 shows 2072 bp fragment of the 100 bp DNA ladder (Invitrogen Canada Inc.). PCR products amplified by primer pairs HSFY-E1F/HSFY-E2R, HSFY-E1F/HSFY-E1R, HSFY-E2F/HSFY-E2R were loaded into lanes 2, 3, 4, respectively. FISH images show the hybridization of the same products in the same order, thus using the whole gene (E), exon 1 (F) or exon 2 (G) specific probes. Images in the upper row (B, C, D) show the metaphase chromosomes with only 4′,6-diamidino-2-phenylindole (DAPI) counterstaining. Bar represents 10 µm.

All three different FISH probes generated an intense, specific, yet dispersed signal on the Y chromosome. The signals were almost chromosome painting probe-like and labeled the majority of the q-arm of the Y chromosome (Figure 6). There were no differences in the hybridization pattern between the probes.

### HSFY expression

**Figure 7:**
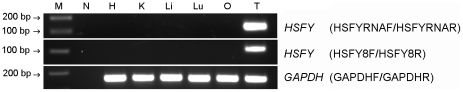
*HSFY* expression in different tissues. *HSFY* was analyzed in a variety of tissues including testis (T), lung (Lu), heart (H), liver (Li), kidney (K) and ovary (O) using real time PCR and two different *HSFY* primer sets to amplify the gene as well as the reference gene, *GAPDH*. The results show that both HSFYRNA (HSFYRNAF/HSFYRNAR) and HSFY8 (HSFY8F/HSFY8R) primer sets amplify *HSFY* only in the testis and no other tissue. All tissues show expression of *GAPDH*. M) 100 bp ladder (Invitrogen Canada Inc.).

An analysis of a variety of different tissues (heart, kidney, liver, lung, ovary and testis) showed expression only in the testis for both HSFYRNA primers (spans intron 1) and HSFY8 primers (specific to exon 1) (Figure 7). *GAPDH* was present in all tissues.

For the bulls for which we had testis tissue samples (n = 22) we measured *HSFY* expression relative to *GAPDH*. We found significant bull-to-bull variation of *HSFY* mRNA levels (p<0.0001). We also found significant correlations of *HSFY* mRNA levels to *UCHL1* (r = 0.4832, p = 0.0227) and *TRPC2* (r = 0.6966, p = 0.0003) which are mRNA markers of spermatogonial and spermatocyte cells, respectively [26], [27]. There was no correlation between *HSFY* copy number and *HSFY* mRNA levels (p = 0.3171).

## Discussion

### HSFY copy number

The sex chromosomes are thought to have originated from a pair of ancestral autosomes and although the X chromosome still remains highly homologous between species, the Y chromosome underwent a series of drastic inversions and species-dependent rearrangements that led to the current Y chromosome being highly heterogeneous in both size as well as in the genetic makeup among species [9], [20], [31]–[33]. Recently it has been shown that even closely related mammals such as humans and chimpanzees show enormous diversity in the MSY of their Y chromosomes for both structure and gene content [9].

One purpose of this study was to characterize the gene copy number of an MSY gene, *HSFY*, in bulls and determine whether or not it varies between individuals as a copy number variant (CNV). Since this study did not address how many of the copies are actually functional, we classify the copies found here under the common distinction of “*HSFY* family”. Since we found no amplification signal in female, we can be sure that the *HSFY* copies we are analyzing are specific to the Y chromosome and are not found on autosomes or the X chromosome (Figure 4).

This is the first study to estimate the number of *HSFY* family gene copies in cattle. We found that there are approximately 73 copies of the *HSFY* family genes on the Y chromosome. Our findings confirm the suggestion that *HSFY* is multi-copied in cattle [20]. The copy number is greatly expanded when compared to what has been reported in humans (2 functional copies, 4 *HSFY*-similar copies (∼82% homology to *HSFY*)) and cat (8 functional copies, unknown *HSFY*-similar copies) [15], [18]. Other species have not yet been characterized.

The fact that we found this gene family to be multi-copied and largely expanded as compared to humans is not unusual for the Y chromosome. *TSPY* is another example of a multi-copy gene that shows expansion in cattle as compared to humans. Cattle have between 37–200 copies, while humans only vary from 20 to76 copies [2], [23], [34], [35]. It is therefore possible that other genes, like *HSFY*, could also show expansions in cattle. Interestingly, the Y chromosome of the domestic cat appears to be made up almost entirely of multi-copied genes and even typically single copy genes such as *SRY* might be present in up to 4 copies [18]. This further demonstrates the extreme heterogeneity of the Y chromosome among species. Not all genes, however, show expansion in cattle. There are some single copy genes present on the bulls' Y chromosome such as *SRY*, *ZFY* and *EIF1AY* that are also found in single copy in humans [20], [36], [37].

We found that the genomic copy number of *HSFY* did not change significantly from bull to bull and therefore it does not appear to exist as a CNV. Deletions of *HSFY* copies in humans have been implicated in cases of male infertility [14], [17], [38], [39]. The bulls in this study had differences in fertilities, as measured by non-return rates (NRRs), ranging from low fertility (NRR = 49.6%) to high fertility (NRR = 77.6%), but regardless of the bulls' fertility status, their *HSFY* copy number stayed relatively constant. The difference may be that humans have only 2 copies of this gene and a deletion in one or both copies can have severe impacts on fertility. Cattle, on the other hand, have about 73 copies of *HSFY* which may allow for many “backups” in case one or more of the copies undergo mutations, thereby minimizing/eliminating harmful effects on fertility.

### Localization of HSFY by FISH

Fluorescence in situ hybridization confirmed the results of the molecular copy number determination and localized it on the Y chromosome for the first time. The largest PCR produced probe, spanning the whole gene (1686 bp) generated an intense hybridization signal which covered almost the whole long arm of the Y chromosome (Figure 6). This suggests amplification of the targeted sequence and the presence of dispersed multiple copies. To test whether the gene specific sequences or an unknown highly repetitive element in the intron caused the observed signal distribution, we amplified the two exons specifically with PCR and used these as FISH probes as well. Both small probes (515 bp and 752 bp) generated the same hybridization pattern (Figure 6), thus we could exlude the contribution of intronic sequences.

### HSFY expression

Notably, we found testis-specific expression of *HSFY* in cattle, as is seen predominantly for human *HSFY* [2], [15]. Both human and feline studies have shown that many multi-copied Y chromosomal genes have acquired testis-specific expression and function so it is not surprising that we also found testis-specific expression of *HSFY* in cattle [2], [19], [40], [41]. It is theorized that the evolution of multi-copied genes in the MSY allows for conservation of genes that enhance spermatogenesis as well as allow for a high level of expression of these genes through gene dosage effects [2], [41]. Since we did not find evidence of a connection between *HSFY* mRNA levels and copy number, there is likely no dosage effects associated with this gene, but it is possible that its multi-copied nature may represent a mechanism of gene conservation. The mRNA levels of *HSFY* varied significantly between bulls (p<0.0001) and correlated positively with mRNA markers of both spermatogonial and spermatocyte cells. This suggests that HSFY is being expressed in spermatogenic cells, as it is in humans, however further immunohistochemical analysis would be necessary to make firm conclusion about its distribution [14].

In conclusion, we found the *HSFY* family to be largely expanded as compared to other species studied to date and it is present in about 70 copies. While it does appear to be a multi-copy gene, it does not appear to be a copy number variant, because the gene copy number did not change within our Canadian Holstein bull sample set of over 20 bulls. FISH results showed that the copies are dispersed across the entire long arm of the Y chromosome. An analysis of mRNA showed its expression is testis-specific, like many other MSY genes, and its expression may be localized to spermatogenic cells as it is in humans.
